# Trans materialist critique as feminist practice: lessons from a polemic against nonbinary identities

**DOI:** 10.3389/fsoc.2025.1646508

**Published:** 2025-10-13

**Authors:** Eric Llaveria Caselles

**Affiliations:** Center for Interdisciplinary Women and Gender Studies, Faculty of Humanities and Education, Technical University of Berlin, Berlin, Germany

**Keywords:** trans theory, nonbinary discourse, gender identity, feminist critical theory, travesti discourse, resignification, neoliberalism, gender politics

## Abstract

This article explores the tensions between trans materialist critique and nonbinary identities, using Kadji Amin’s essay “We Are All Nonbinary: A Brief History of Accidents” (2022) as a point of departure. It asks two guiding questions: How should we understand the relationship between trans and nonbinary identities? And what kind of trans materialism can meaningfully respond to our current political moment? The article focuses on three problematics: (1) the reliance on abstract philosophical critiques of gender identity; (2) the framing of trans and nonbinary identities as objective versus subjective, respectively; and (3) the portrayal of nonbinary identity as emblematic of neoliberal ideology. Drawing on posttranssexual (Susan Stryker, Sandy Stone), travesti (Lohana Berkins, Marlene Wayar), and nonbinary (Eris Young, Travis Alabanza) narratives—as well as critical feminist theory (Judith Butler, Nancy Fraser, Seyla Benhabib, Regina Becker-Schmidt)—the article argues for understanding gender identity claims as situated political practices that are simultaneously subjectively and objectively constituted. It critiques positivist tendencies within trans materialism and challenges polarizing distinctions between trans and nonbinary identities. Ultimately, it builds on Wendy Brown's definition of neoliberalism to argue that dismissing nonbinary identities on materialist grounds risks constructing the trans critic as a neutral subject outside power, becoming another site for the hollowing out of democratic practice. The article calls for a trans materialism grounded in self-critical feminist analysis and relational political practice.

## 1 Introduction

### 1.1 Nonbinary disruptions

The rise in popularity of nonbinary identities became a disruptive fact in my life in two different ways^1^. On a private level, the continuous encounters with people who identified as nonbinary made me question the terms in which I understood my gender identity. My decision to socially and medically transition was enabled by a trans culture that taught me that I did not have to understand these decisions as a return to any intrinsic masculinity. I had never felt at home in womanhood, and I did not expect I would find a home in manhood. I learned about trans as a queer gender, outside of the categories of “man” and “woman,” and not necessarily bound to a binary medical transition. However, the use of “nonbinary” suggested to me that this meaning of “trans” has been lost or partially displaced. Am I nonbinary now? What keeps me from just calling myself so? Are trans and nonbinary identities politically equivalent?

In feminist scholarship, I am invested in the current trans materialist turn. I situate myself within a trans-theoretical current in the Western context that draws from diverse historical materialist and Marxist-feminist references to critique hegemonic trans discourse and elaborate alternative conceptualizations and narratives (Clochec and Grunenwald, 2021; Gleeson and O’Rourke, 2021; Terán and Travis, 2024). Despite its internal heterogeneity, I want to point to three common threads that are relevant to the discussion of nonbinary identities. First, the materialist turn takes issue with a notion of transgender identity as an individualized sense of self and the foundation of trans politics. Instead, materialist approaches emphasize the embeddedness of trans becoming in social relations of reproduction, defining being trans as a practical and collective accomplishment rather than a subjective feeling. Second, trans materialist approaches shift the focus in narratives of violence from identity misrecognition to experiences of precarity in employment, housing, healthcare, etc. Finally, trans materialism defines itself through a vehement critique of liberal trans rights strategies and urges a rethink of trans politics as intersectional and revolutionary class politics.

When I first read Kadji Amin’s “We Are All Nonbinary: A Brief History of Accidents” (Amin, 2022), I felt like the trans materialist take on nonbinary identities that I was looking for had been written. In it, Amin situates nonbinary identities as the epitome of a historical process of proliferation of gender and sexual identities in which critical meanings are displaced by a tendency to idealize and reify gendered and sexual identities. Nonbinary discourse, Amin writes, “has taken gender self-identification far further than trans people even envisioned” and “doubled down on the notion of gender as an internal psychic identification, adding the corollary that nonbinary identification is “valid” regardless of outward expression” (114–115). Amin understands nonbinary discourse as a product of “the neoliberal universalization of identity as the basis of all politics” (115) that has introduced a concept of gender identity “autonomous from the social” (116). His judgment is scathing:

In this sense, contemporary gender identity is the apotheosis of the liberal Western fantasy of self-determining “autological” selfhood (…). It is therefore difficult to imagine an identity more provincially Western and less decolonial than contemporary nonbinary identity. (116)

For Amin, a central reason to engage in this analysis is to “consider the harms that the coinage and idealization of normative identities (…) has wrought on ordinary gender-variant people, particularly trans femmes” (107). Amin argues, for example, that a rise in transmisogynistic violence “may therefore be a consequence of the homo/hetero divide” (111) and that feminine gay men have become “fallouts” and lost any “affirmative term to identify them” as the product of the homo/hetero and cis/trans divide (112). In line with the commitment to a fundamental critique of gender and sexual identities, Amin advocates “abandoning Western binary and taxonomic thinking” (117) to pursue “a robust trans politics and discourse *without* gender identity” (118). At the same time, he also argues that “it might be necessary to generate new identities, given that nonbinary is not a true social category” (117). Although Amin’s point of view initially strongly resonated with my intuitions, it eventually became clear to me that his piece was not the end of the nonbinary disruption but rather the beginning of unsettling my understanding of trans materialism.

### 1.2 Unsettling trans materialism

Following my discomfort with Amin’s take while holding on to my commitment to trans materialist theorizing, I came to three theoretical and political problematizations. First, I was not sure of the implications of the philosophical critique of gender identity on the terrain of trans/nonbinary politics. I was suspicious of a trans materialist critique that presents itself as a judgment about how people should or should not identify, making claims on the validity of the identification of individuals: why would a trans dismissal of nonbinary identities be acceptable when the dismissal of trans identities is not? Is there a trans materialist understanding of gender identities, or is the concept itself inimical to a trans materialist standpoint? How can we make claims on the harms and benefits of different gender identity politics? Second, is it possible to establish a distinction between trans and nonbinary identities along a separation of objective vs. subjective, real vs. unreal? What understanding of materialism and of social phenomena supports such a categorical and normative distinction? And what are the political implications of dismissing the subjective as unreal, not a field of struggle for materialist politics? Finally, on what basis can it be claimed that nonbinary identities are more a product of neoliberalism than other (trans) identities? What understanding of neoliberalism underlies this claim? And what are the implications of being declared a participant in neoliberal logics?

These are the three problems that I address in this article, which bind together the question “what is the relation between trans and nonbinary identities?” with the question “what defines a trans materialist approach that can make a relevant contribution to our present?” For the first question, I draw from a close reading of selected posttranssexual (Susan Stryker and Sandy Stone), travesti (Lohana Berkins and Marlene Wayar), and nonbinary (Eris Young and Travis Alabanza) texts. My selection follows theoretical and political purposes in that it allows for reopening two central distinctions from Amin’s text, namely, the distinction between anti-identitarian radical politics and identitarian assimilationist politics, and the distinction between a definition of trans politics as being based on an embodied notion of transition in contrast to nonbinary politics as being based only in reference to identification and disregarding embodiment.

For the second question on how to define a trans materialist approach, I take on the three theoretical problems outlined previously: (1) the implications of the critique of gender identity, (2) the distinction between objective and valid identity categories versus subjective and questionable identity categories, and (3) the understanding of neoliberal ideology and its impact on gender identities. The article is structured around three key issues.

In the first section, which deals with the critique of gender identity, I draw on Judith Butler’s elaboration of the limitations of philosophical critique, Nancy Fraser’s understanding of social identities as political practices, and Seyla Benhabib’s dialogical model of identity to define a critical materialist approach to analyzing gender identity claims. Based on this understanding, I read posttranssexual and travesti political projects to substantiate the claim that a trans materialist critique of gender identity should not derive political implications merely from abstract philosophical principles but rather pay attention to the conditions of possibility for political projects and hold space for the way they become meaningful and effective in ambivalent ways.

In the second section, I address Amin’s distinction between valid identity categories based on objective criteria (trans) and questionable identity categories based on intangible, subjective criteria (nonbinary). To this end, I build on the German feminist critical theory tradition to reintroduce a notion of social phenomena as simultaneously composed of objective and subjective dimensions. This allows me to read posttranssexual and travesti discourse as a feminist analysis that attends to this contradictory constitution of gender politics. I then move on to investigate the kinds of social relations that are figured in nonbinary discourse. I present a reading of Eris Young’s narrative in “They / Them / Their” as something close to Amin’s characterization of nonbinary politics, which I conceptualize as a privatization of gendered interpellation rather than as a lack of positive social content or elimination of transition, as Amin does. I then introduce Travis Alabanza’s memoir, “None of the Above,” as a counterexample to Young in nonbinary discourse, which embodies a critical and complex notion of autonomy.

In the third section, I examine the claim that nonbinary identities represent a neoliberal capture of more radical trans politics, drawing on Wendy Brown’s definition of neoliberalism as a hollowing out of democratic structures (Brown, 2015). I argue that Young’s and Alabanza’s nonbinary imaginations of freedom and political belonging are animated by an identification with political purity that leaves little space for legitimate contestation and contrasts with posttranssexual and travesti politics. Finally, I turn the question toward the trans critic to raise the claim that not only nonbinary discourse but also trans materialist critique can be a site from which neoliberal ideology gets perpetuated. It is precisely through the narrowing down of the meaning of materialism and the marginalization of important contributions from feminist theory that the voice of the trans critic is constructed as untouched by neoliberal ideology and presents itself as a source of redemption narratives. In the conclusion, I reclaim a trans materialist that incorporates a situated and critical self-interrogation of the conditions of one’s own thinking and the functions of its narratives.

## 2 On the critique of gender identity

Amin builds his argument against identity politics based on Butler’s early critique, which he characterizes as “a caution against any faith in the purity and distinctness of identity categories” (Amin, 2022, 107). His genealogy of the conceptual separations between heterosexuality and homosexuality, between trans and cis, and, finally, between nonbinary and binary, suggests a causal relation between these processes and harms “on ordinary gender-variant people, particularly trans femmes” (*Ibid.*)^2^. Amin calls for trans discourse to “develop a tolerance for contamination and for the inevitable misfit of identity categories” and to abandon the “impossible dream … of social categories capable of matching the uniqueness of individual psyches” (117). For him, “this means developing a robust trans politics and discourse *without* gender identity” (118).

### 2.1 The limits of philosophical critique

I want to begin my examination of Amin’s disavowal of gender identity in trans politics and discourse with a re-reading of Butler’s critique of gender identity, which pays attention to the implications that Butler derives for political practice, as articulated in the debate with Benhabib et al. (1995). As Amin correctly points out, Butler argues that feminist critiques need to account for the fact that to assume an identity necessarily relies on exclusion. However, for Butler, this insight does not mean “to negate or to dismiss, but to call into question” the subject of identity (Butler, 1995a, 49). The argument is thus not that gender identity claims are per se to be rejected, but that the necessity of acquiring a subject position and speaking from it needs to be reconciled with another necessity of feminism: to interrogate the normative and exclusionary implications of identities.

This is a crucial point for trans theory, since the trans subject emerges as the abject of the binary heteronormative gender order and is thus constantly engaged in the activity of trying to make sense of itself, without fully succeeding. Gender identity claims enacted by trans and nonbinary subjects are, in a sense, always engaged in a process of disruption of the hegemonic rules that confer gendered subjectivity. The mere fact that we have to articulate a gender identity as individuals in our daily lives is part of this process of resignification, even though the agency at the level of resignification does not correspond with our individual intentionality or the practical purposes of our actions.

However, what implications does Butler derive from the need to interrogate the exclusionary implications of identities for political practice? More specifically, can we raise normative demands that must be met by trans and nonbinary identity claims? The single substantial demand is the rejection of forms of identity that rely on abjection, where the disavowal of the other is a condition of the “I” or the “we” (Butler, 1995b, 140). Beyond this normative commitment, Butler does not offer substantive criteria by which to settle which concrete forms of identity can be deemed better or worse, more or less politically valuable. In fact, Butler’s point is precisely that the belief that political conflicts can be solved on philosophical grounds is a fundamental mistake:

The claim that every political action has its theoretical presuppositions is not the same as the claim that such presuppositions must be sorted out prior to action. It may be that those presuppositions are articulated only in and through that action and become available only through a reflective posture made possible through that articulation in action. (Butler, 1995b, 129)

Butler’s argument here belongs within a trans materialist framework that challenges the idealist misconstruction of political conflicts as philosophical debates or reduces political struggles to theoretical questions. A trans materialist critique that disqualifies trans or nonbinary identity politics on abstract philosophical grounds is ignoring this. Instead, I argue that a trans materialist analysis needs to understand that the resignification that trans and nonbinary identity projects enact, even if they can be read philosophically in relation to the macrological plane of the gender order, are pragmatic acts of struggle for existence in a historical reality and political situation outside of the control of an insurgent minority. The refusal to set substantive criteria for identities is a call to the feminist critic not to fall into “its own authoritarian ruse” by “establishing a normative foundation for settling the question of what ought properly to be included in the description of women” (Butler, 1995a, 51), or, in this case, trans or nonbinary identities. Based on Butler’s articulation of the limitations of philosophical critique as grounds for a critique of political practice, I argue that to establish a materialist case about the benefits and harms of trans and nonbinary political projects, we need to approach the level of historical processes and political struggles. In order to do so, we also need a conceptualization of gender identity as an object of critical social analysis.

### 2.2 Gender identity claims as political practices

Drawing on Nancy Fraser, I understand gender identity as a set of political claims, that is, as practices embedded in struggles to exercise control over and shape gender relations through processes of (re)signification. Against the dominance of psychological frameworks in feminist analysis of gender identity, Fraser argues that gender identities “are discursively constructed in historically specific social contexts; they are complex and plural; and they shift over time” (Fraser, 1997, 152). In this perspective on gender identity claims, what matters less is the individual sense of self but rather the power-laden processes by which groups of people come to adopt specific notions of gendered subjectivity and the implications of their cultural and institutional recognition. In Fraser’s words: “How does it happen, under conditions of inequality, that people come together, arrange themselves under the banner of collective identities, and constitute themselves as collective social agents? How do class formation and, by analogy, gender formation occur?” (*Ibid.*, 153). In this meaning, we can “see” gender identity not just in the articulation of a feeling of gendered belonging that only trans people seem to have (Amin, 2022, 113f.), but equally in the claim that to be a woman means to be born with certain genitalia or reproductive organs.

I believe that Seyla Benhabib’s dialogical narrative model of identity construction is a further helpful reference for a discourse analysis of gender identity claims as political practices. For Benhabib, “to be and to become a self is to insert oneself into webs of interlocution; it is to know how to answer when one is addressed; in turn, it is learning how to address others” (Benhabib, 1999, 344). Benhabib seeks to emphasize that, “although we do not choose the webs in whose nets we are initially caught (…) our agency consists in our capacity to weave out of those narratives and fragments of narratives a life story that makes sense for us, as unique individual selves” (*Ibid.*). Benhabib’s notion of agency allows us to acknowledge intentionality and autonomy in the individual process of becoming a self but also establishes that narratives of the self cannot have closure because “the sense that I create for myself is always immersed in a fragile “web of stories” that I, as well as others, spin” (Benhabib, 1999, 348). In this theoretical framework, gender identity claims are stories we tell ourselves and others. While we do not choose the narratives or the web of interlocutions we are thrown into, we have the capacity to resignify them in the process of making sense.

In the next two sections, I develop a reading of the posttranssexual discourse of Sandy Stone and Susan Stryker and of the travesti discourse of Lohana Berkins and Marlene Wayar as gender identity claims. Stone and Stryker’s posttranssexual project emerges in the context of US urban milieus, specifically queer academic, artistic, and sexual subcultural spaces in the late 1980s and 1990s, under the impact of postmodern and poststructuralist theory. The writing of Berkins and Wayar emerges from the 2000s on as a way to document, articulate, and critically reflect on the political struggles of travestis in Argentina.

These discourses are relevant for revisiting Amin’s problematization of identity politics because they articulate posttranssexual and travesti challenges to the dehumanization of transsexuals and travestis that problematize the demand for fixed meanings itself. At the same time, they push for political projects such as the establishment of Trans Studies as an academic field or the Gender Identity Law, which stand in tension with the radical horizon of identity critique. This reading thus reopens the notion that radical anti-identitarian and assimilationist identitarian politics are separable and questions whether radical anti-identitarian politics are always practicable or inherently more beneficial.

My selection of these authors also challenges two further a *prioris* in Amin’s analysis. First, by including the travesti movement in Argentina, I question the appropriateness of an analysis that only considers the Anglophone West and ignores the global impact of Southern trans politics. Second, by selecting trans women and travesti authors, I open up Amin’s claim that trans women should be seen as the victims of the separation between sexual and gender identities (Amin, 2022, 111–112). While I am not arguing with the documented higher levels of violence experienced by trans feminine groups compared to other gender identities, I think it is important to acknowledge the agency of trans feminine subjects and to give space to their analysis when making normative claims on their behalf.

### 2.3 Posttranssexual and travesti resignifications

There is a statement by Stryker that encapsulates the resignification of transsexual identity that she and Stone aimed for: “I name myself a transsexual because I have to, but the word will mean something different when I get through using it. I will be a new kind of transsexual” (Stryker, 1998, 152). Stryker’s formulation makes clear that her use of the term “transsexual” involves a form of coercion: there is no choice in self-naming as transsexual, and no choice in the meaning of the term or the implications of being named as such (Stone, 2006; Stryker, 2006, 2024a). However, what kind of resignification do Stone and Stryker propose? Focusing on the symbolic dimension of this normative order, Stones advocates a posttranssexual practice that she defines as a refusal of passing in order “to be consciously “read,” to read oneself aloud—and by this troubling and productive reading, to begin to write oneself into the discourses by which one has been written—in effect, then, to become a (look out—dare I say it again?) posttranssexual” (Stone, 2006, 232).

The posttranssexual resignification intervenes within a scene of interpellation, whereby Stone and Stryker refuse the terms in which they are called. They do not seek to establish a new, clear meaning but to challenge the belief in categorical boundaries and show the violence of the fixation of meaning. Their notion of transsexuality is a queer one that “represents the prospect of destabilizing the foundational presupposition of fixed genders upon which a politics of personal identity depends” (Stryker, 2024a, 135). In this regard, Stone and Stryker’s project clearly aligns with Amin’s call “to develop a tolerance for contamination and for the inevitable misfit of identity categories” and “to relinquish the fantasy that gender is a means of self-knowledge, self-expression, and authenticity” (Amin, 2022, 117).

Berkins and Wayar also undertake a project of resignification. Their point of departure is the term “travesti” in its prevalent negative meaning, a synonym of “sidosa, ladrona, escandalosa, infectada, marginal^3^” (Berkins, 2012; see also Wayar, 2019, 23). These meanings are not rejected as falsehoods but challenged in their dehumanizing effect and the ways that they legitimize and depoliticize the violence in travesti’s lives. As Wayar writes: “es una interpelación compleja ante una misma, ante la sociedad, de decir: soy esto, en qué medida me lo vas a respetar?”^4^ (Wayar, 2019, 22). Identity, for Berkins, is not a given or an essential quality, but a political consciousness, “una manera de vernos y ser vistas de una manera que puede permitir o impedir el reconocimiento, el goce, el acceso a derechos^5^” (Berkins, 2013). Berkins’ and Wayar’s stories of mutual support, joy, and intimate friendship, of disputes, pain, and hurt, are populated by concrete individuals, memorializing travesti life and community against the anonymity and impunity of their violent deaths. This narrative humanizes travestis and constructs them as political subjects, inscribes their struggles in a larger narrative of progressive popular movements, and interpellates the state to recognize them.

At the same time, Berkins and Wayar refuse to affirm unconditionally travesti culture. Berkins writes of travestis as contradictory subjects, riddled with paradox and tensions (Berkins, 2012), “atravesadas por la superficialidad del mercado”^6^ (Berkins, 2007). She and Wayar critique that travesti expressions of femininity are defined by patriarchal ideas and comment on the heteronormativity, individualism, lack of solidarity, political disinterest, or racism of travesti communities (see Berkins, 2004; Wayar, 2019; Álvarez and Fernández, 2021). Their project of defining travesti as a political identity contains an intra-community interpellation to unlearn “nuestra parte opresora”^7^ (Berkins, 2003) and develop new desires:

Cuando les preguntas en un taller qué quieren ser, te contestan: travesti. O mujer. Quedan atrapadas en esa ficcionalidad, y en esa cosa de ser sólo travestis. Recién después de mucho trabajo salen otros deseos: maestra, bailarina, médica^8^ (Berkins, 2007).

Although anchored to a notion of identity, the travesti politics of Berkins and Wayar operate precisely on an understanding of identities as sites of transformatory political practice that can be read in alignment with Amin’s critique of the idealization of identities.

### 2.4 Calculus of harms and benefits

Amin claims that the reification of gender identities has meant a capture of critical meanings and has marginalized transfeminine people. His level of historical abstraction, however, does not allow us to understand how this reification is accomplished and how, ultimately, these harms could have been prevented. Amin reads the neoliberal capture of gender and sexual politics as the culmination of four logics: “*idealization,*” “*divergence*,” “*binarism*,” and “*autology*” (Amin, 2022, 107; emphasis on the original). It remains unclear, however, what these historical logics that Amin proposes represent at the level of concrete political practice. A close analysis of posttranssexual and travesti politics can help shed light on the difficulties of calculations of harms and benefits mapped on a distinction between liberal identitarian or radical anti-identitarian logics. Drawing on the authors’ analysis, I question the assumption that following a radical post-identitarian politics is always practicable and inherently beneficial for “ordinary gender-variant people” (Amin, 2022, 107).

As Stone and Stryker explain, their radical project acquired its practical plausibility from a context in which postmodern and poststructuralist critique was cultivated within the institutional landscape of the humanities and social sciences, as well as in art spaces and sexual subcultures: “We felt like we were reinventing the world, reinventing family, reinventing love, reinventing ourselves.” (Stryker, 2024d, 80; see also Stone, 1995, 165). However, as Stone remarks, the economic, cultural, and political conditions could not be kept indefinitely, limiting the utopian radicality to “this brief time of upheaval and promise (…) before the long night sets in and such strategies are no longer possible” (Stone, 1995, 166).

However, even in these conditions, who could access such a radical political practice? For whom was it relevant? The posttranssexual resignification depends on a form of authorship acquired in proximity to elite academic institutions, far away from the majority of transsexual women. As Namaste’s critique makes clear, the posttranssexual project has a difficult translation into the needs and lived realities of the marginalized sectors of trans populations and does not seem conducive to the improvement of their living conditions (Namaste, 2005; Valentine, 2003). However, even for those who believed in its political necessity, the posttranssexual resignification does not feel like a state of harmony but is a permanent struggle with the reality and the grip of hegemonic meanings inscribed in our bodies, desires, and relations (Stryker, 2024b).

In addition, I think it is important to consider that if we can retrieve the posttranssexual critique, it is partially because of the institutionalization of Trans Studies. The claim that the posttranssexual ethos in Trans Studies has been overwritten by a mainstreaming of transness (Stryker and Wark, 2024, 173) or debates on whether Trans Studies has failed to live up to its potential (Chu and Drager, 2019; Adair et al., 2020) are dependent on a value and relevance of trans critique acquired in part through its harnessing of the academic apparatus and the hegemonic position of the United States. This exemplifies that the possibility to reflect on the losses of critical horizons might be enabled precisely by the same processes that are blamed for the loss. A serious assessment of the benefits and losses of political projects cannot necessarily be fitted into either-or, for-or-against propositions of identitarian and post-identitarian politics.

This point is more dramatically illustrated in the case of Berkins and Wayar. Wayar’s recent writing expresses a sense of political loss that echoes that found in Amin’s critique. In a dialogue with activist and artist Susy Shock, they mourn the death of leaders of the travesti movement, such as Berkins, and establish a connection between these deaths and problematic tendencies in current activism (Wayar, 2021, 76; see also Wayar, 2019, 110–111). However, this sentiment needs to be contextualized in “the difficulty of political life” (Butler, 1995b, 131) and the discrepancies between travesti activists pursuing different interests and strategies (Álvarez and Fernández, 2021). In this complicated and heterogeneous context of travesti politics, Wayar and Berkins threaded a tension between, on the one hand, an interpellation to society and the state from a travesti standpoint to demand recognition and inclusion and, on the other hand, a self-critical interpellation to travestis from a feminist standpoint to challenge their attachment to oppressive norms. A case pertinent to the problem of the reification of identities in which this tension came to the fore was the struggle for a gender identity law.

One decision they had to make was whether the law would define in a binding manner who counted as transsexual, travesti, or transgender and could access the possibility of legal gender change (Fernández, 2020, 169; Berkins and Ernesto, 2012). Berkin’s rejection of this option was part of her anti-essentialist notion of gender and her commitment to holding space for the openness of future gendered subjectivities. Instead, the notion of “gender identity,” imported from the Yogyakarta Principles through the inter and trans activist Mauro Cabral, became a pragmatic tool to translate this political vision into the legal architecture of the state and reconcile it with the demands of recognition and inclusion (Fernández, 2020, 170–171). The instrumental character of the category of “gender identity” in the interpellation of the state and its separation from a notion of identity as ungovernable was explicit:

Si bien necesitamos anclar la identidad, de alguna manera, para interpelar a los Estados en busca de políticas públicas de inclusión positiva, también debemos tener en claro que en lo cotidiano la identidad es un concepto no universalizable, no uniformable. (Wayar, 2009, 3)^9^

When the gender identity law was passed in 2012, Wayar reached out to Berkins, expressing her concerns that the law would mean the end of the project of a political travesti identity in its utopian horizon (Fernández, 2020, 175). Berkins did not dismiss Wayar’s concerns but insisted on the necessity to pursue a politics that could tangibly ameliorate the living conditions of travestis (*Ibid.*). Berkin’s response to Wayar raises important objections to the underlying normative assumptions of Amin’s critique. What would have been the harm of refusing to engage in the struggle for legal self-determination because it contributed to the reification of gender identity? What concrete benefits would have been lost through a radical attachment to a trans politics without gender identity?

The analysis of the situated political struggle highlights the practical challenges of sustaining radical utopian projects, ultimately revealing a tension between a deconstructivist critique and the political demands relevant to the needs of actually existing subjects. In my view, this suggests that trans materialist critique needs to situate itself within this tension and acknowledge the complicated co-occurrence of assimilationist and radical politics, including the limits of the latter.

## 3 On the relation between trans and nonbinary identities

### 3.1 Positivist tendencies

Amin’s analysis constructs a delimitation between trans and nonbinary identities, according to which “nonbinary discourse” has “taken gender self-identification far further than trans people ever envisioned” (2022, 114). This is because “nonbinary might “look” any number of ways and need not find external expression in choice of dress, hairstyle, pronouns, or any other social marker of gender” (*Ibid.*). For Amin, nonbinary discourse “has doubled down on the notion of gender as an internal, psychic identification, adding to the corollary that nonbinary identification is “valid” regardless of outward expression,” while trans discourse was bound to the desire to have medical or social transitions validated (114–115).

Amin connects this distinction between a nonbinary discourse centered on identification and a trans discourse centered on transition to the question of what “should be the basis of gender categorization at all” (115). He replies that gender categories should rely on socially relevant aspects: “What is socially relevant is *transition* – a shift in social gender categories, whatever they might be – not *identification* – a personal, felt, and thereby highly phantasmatic and labile relation to these categories” (115). In a contradictory move to his call for trans politics without gender identity, Amin proposes here to create “one or more socially legible gender categories—based on presentation and behavior, not self-identification alone—for those who want to transition from men or women to something else, something with positive social content rather than something devoid of it, as nonbinary currently is” (116).

I read Amin as advocating a positivist epistemology from which supposedly better, more relevant political categories could be proposed. The desire to ground trans politics in tangible social reality, often equated with embodied gender transitions, is not unique to Amin and can be found in proponents of trans materialist approaches (Clochec, 2023; Gill-Peterson, 2024). Considering the intensified denial of trans identities in general public discourse and the insufficient attention to the economy-based dimension of trans oppression (poverty, housing precarity, lack of access to formal employment, healthcare, etc.) in liberal trans politics, I see strategic reasons for trans political projects that link gender identity categories with observable forms of embodiment and corporeal practices and quantifiable definitions of oppression. From a theoretical standpoint, however, the distinction between the *objective-observable-real* and the *subjective-intangible-ideological* as the proper objects of social analysis and political struggle belongs to a positivist framework that stands in tension with the historical materialist tradition.

In the previous section, I defined gender identity claims as political resignification practices that allowed for the interrogation of the conditions of possibility and contradictions of identitarian and anti-identitarian trans political strategies. In the next sub-section, I draw from German critical gender theory to argue that gender identity categories need to be understood as both objectively and subjectively constituted social entities.

### 3.2 Gender identity within a critical concept of the social

According to Tanja Paulitz, the German feminist problematization of the category “woman” in the 1980s and 1990s differs from the US one in that it did not rely on the distinction between sex and gender but rather on a development of the conceptual tools from the early critical theory to define women-subjects in their historical and social specificity (Paulitz, 2019, 392). This implied a project of feminist theory centered on the analysis of gender relations within a concept of society as a historical and interrelated totality and an understanding of social critique as a self-reflected, situated endeavor (Knapp, 2023, 38–45). A theorist within the tradition, whose work is directly relevant to the theoretical question at stake, is Regina Becker-Schmidt. She is most known for her notion of “doppelte Vergesellschaftung”^10^, which she developed to describe the structural position of women based on studies of the experiences of working mothers in the Federal German Republic in the 1980s (Becker-Schmidt, 2010). That term identifies a dilemma that characterizes women’s social location as well as psychosocial biographies, as they are expected to participate in both household and formal work, two social spheres with contradictory logics (*Ibid.*). The strain that women experience is symptomatic of both the separation of reproductive and productive labor and the polarization and hierarchization of genders. At the same time, this social arrangement is stabilized by the privatization and naturalization of the conflicts that women experience (*Ibid.*).

This analysis stands for Becker-Schmidt’s definition of the central task of social critique, namely, the reconstruction of “Vermittlungen,” that is, both the interrelatedness of entities that are separated structurally and ideologically and the operations by which the separation is upheld and the purposes it fulfills:

Wir stoßen in unserer Kultur auf einer Reihe gängiger Vorstellungen, die Bezogenes entgegensetzen: Geist/Stoff, Intellekt/Körper, Natur/Kultur, Subjekt/Objekt, Theorie/Praxis (…). Es liegt auf der Hand, dass Denkmuster, die solche Interdependenzen vernachlässigen, eine soziale Funktion haben, deren Problematik sich erst durch Gesellschaftskritik erschließt.^11^ (Becker-Schmidt, 2017, 122)

Becker-Schmidt applies this framework to the distinction between masculinity and femininity and makes the point that dichotomous gender categories are double entities in the sense that they are at the same time real and unreal:

Einerseits müssen sie als Resultate von Geschichtsverläufen verstanden werden. In diesem Sinne sind sie Symptome von Aufspaltungen, die soziale Zusammenhänge zerstören. Andererseits beruhen sie auf Täuschungen: Sie suggerieren zum einen durch Klischeebildung Eindeutigkeit, und sie halten zum anderen Fügungen verdeckt, die das Polarisierte falsch verknüpfen. ^12^(*Ibid.*, 124)

An understanding of gender identities as social phenomena, both objectively and subjectively, has important implications that lead us away from Amin’s positivist tendencies. First, it allows us to conceive of emancipatory political projects as containing what appear to be internal contradictions. Such contradictions become rational in the recognition of the dissonances in the simultaneous operations of gendered structures and norms at different social scales within a historical moment (*Ibid.*, 125). In the next subsection, I complicate Amin’s reading of trans discourse as centered on observable forms of gender transition by presenting posttranssexual and travesti discourse as a complex binding of objective and subjective moments in their social analysis and political proposal.

The second implication from a notion of gender identity as both objective and subjective is that Amin’s characterization of nonbinary identities as lacking positive social content becomes a contradiction in terms. This reopens the task of defining nonbinary identities as social entities: what kind of social content do they represent? In the sub-sections “Nonbinary Autonymy” and “Complicating Nonbinary,” I engage with Young’s (2020) book “They / Them / Their” and Alabanza’s (2023) “None of the Above” to address this question exemplarily^13^. While I interpret Eris Young’s nonbinary politics as a privatization of gendered interpellation, Alabanza’s nonbinary politics carve out a complex meaning of autonomy in line with critical feminist theory.

Finally, from a critical theory standpoint, constructions that rely on a splitting of self and other are understood as serving some ideological function, which is contrasted with the task of critique as a self-reflected and situated practice. In the final section of the article, I address Amin’s question about the neoliberal capture of gender politics to interrogate not only nonbinary discourse but also trans critique.

### 3.3 Corporeality and subjectivity in trans politics

Against Amin’s reading of trans discourse as centered on a positivist notion of transition, which he seeks to valorize, I turn to posttranssexual and travesti discourse as examples of feminist critical analysis. In their definition of transsexual and travesti identity categories, their referent cannot be reduced to a gender transition or a specific embodiment. In both cases, the identity terms bind a corporeality to a subjective element, namely the expression of an individual creativity that refuses to be contained by existing gendered meanings.

In the Posttranssexual Manifesto, Stone uses the term “transsexual” to refer narrowly to those who “wanted surgery” (Stone, 2006, 230). The choice of wording, however, already points to the difficulties of isolating a meaning of transition from subjective feelings: it is not surgery itself, but the desire for it that Stone centers here. What is ultimately relevant in her analysis is not the embodied experience of transition, but how its hegemonic narrativization fixates transsexuals as abject subjects and reproduces patriarchal and heteronormative meanings. Stryker can be read similarly. She claims that “the transition from one sex to another is the single experience that no one other than transsexuals will ever have. Having that experience makes you one of us.” (Stryker, 2024b, 38). However, what is politically relevant about transition, according to Stryker? When approaching her genital surgery, Stryker decided to film the procedure as part of an art project: “I want to see exactly how far I can push a claim — that I’m changing the shape of my genitals and secondary sex characteristics for esthetic and artistic reasons, not because I am eligible to receive a DSM-IIIR diagnosis of 302.5(c) gender identity disorder” (Stryker, 2024c, 42). Stryker joins Stone’s trans political project to disrupt the meanings that seem to naturally flow from objective, physical processes, a disruption that emerges in a creative intervention at the level of narrative and identification.

Berkins and Wayar define travesti as “una identidad encarnada”^14^ (Berkins, 2012), and their reconstruction of travesti lives directs the reader to the corporeal inescapability of social isolation, police beatings, abuse by clients and family members, silicone injections, use of drugs, hunger, and death (Berkins, 2007; Wayar, 2019). These are the realities at the core of the travesti politics and the basis of Berkins’ pragmatism. But Berkins and Wayar’s travesti politics also articulate a notion of gender identity as a struggle for individual autonomy and freedom. For them, the politically relevant aspect of travesti identity is that it contains a moment of unforeseen agency that exceeds available meanings: “pensamos que es posible construir un género propio, distinto, nuestro^15^” (Berkins, 2003). Berkins’ discourse renders visible that travesti identity holds a moment of individual autonomy, which needs to be defended as “una actitud muy íntima y profunda de vivir un género distinto del que la sociedad le asignó a su sexo” (Berkins, 2003, 9; see also Wayar, 2019, 33–38). This moment of individual autonomy and openness goes deeper than a defense of an existing travesti corporeality, a complicated site that cannot be separated from conditions of marginality and oppression, which Berkins does not want to reify:

Pero aun si no hubiese podido acceder a esa transformación, lo mismo yo sería Lohana Berkins. Hoy sé que si yo mañana me saco las tetas y me corto el pelo, sigo siendo Lohana Berkins. No podemos creer que sólo puedes ser travesti con ese cuerpo. (Berkins, 2007).^16^

The notion of travesti identity that Berkins defends here surprisingly resembles Amin’s characterization of nonbinary discourse, reclaiming identification, personal feelings, and political convictions as a foundational moment. His positivist dismissal of identification thus erases precisely the utopian element in posttranssexual and travesti discourse and their politicization of subjectivity. It seems to me that a positivist foundationalism that Amin seems to advocate is a false solution to a political and epistemic problem in that it limits our capacity to understand gendered oppression and to generate socially relevant liberatory meanings.^17^

### 3.4 Nonbinary autonymy

Can we, despite the mischaracterization of trans discourse in Amin’s analysis, claim a distinction between trans and nonbinary identities, by which nonbinary identities stand for “a core of selfhood that requires no expression, no embodiment, and no commonality” (Amin, 2022, 116)? And if such logic for gender identity categories cannot be defined as a lack of positive social content, what kind of positive social content does it then represent? It is certainly possible to find narratives that roughly correspond with Amin’s definition of nonbinary discourse. I read Eris Young’s book “They / Them / Their,” a resource on nonbinary identities for those “who want to understand but might not have the means to do so” (Young, 2020, 8), as one such example. The fact that Young identifies as both trans and nonbinary and that the book contains an extensive discussion of medical transition suggests that its specificity cannot be defined as an elimination of transition, as Amin suggests.

Based on the glossary definitions and Young’s characterization of their own gendered sense of self (12–13; 18), their notion of nonbinary identity refers to an individual affective pattern of disidentification with hegemonic gender categories, which is articulated through an internal ontology of “being” and its expression. Young writes that for nonbinary people, “identity and expression are intimately connected. We express our genderqueer status visibly through our bodies, hair, makeup, clothing, and mannerisms.” (112). However, there is no specific element, style, or substantial relation between identity and appearance (31). There is also no clear relation between nonbinary identity and medical transition or body characteristics (181). Whether the body is relevant to gender identity falls within the realm of individual definition: “A nonbinary person may see their gender as something separate from their body, or they may not see the point in changing anything about their body because their gender is expressed in their speech or behavior, rather than their appearance” (182). The same applies to the relationship between nonbinary gender identity and the use of pronouns.

The only marker for nonbinary identities in Young’s definition is the individual act of self-categorization: “There are multiple separate identities encompassed within the larger category of “nonbinary,” and there is no single way to be nonbinary, either” (52). At the individual level, Young’s definition of nonbinary is about having the moral decency to address people in ways that recognize their sense of self (58). However, at a collective level, nonbinary recognition necessitates a primacy of individual meanings over the need for socially shared categories. In that sense, nonbinary identity stands for something other than an identification beyond “man” and “woman,” and something other than the elimination of transition. Instead, I propose understanding nonbinary identity in Young’s sense as a project of privatizing gendered interpellation, which privileges language as a site of gender politics. Young, a linguist by training, offers a useful concept to characterize this form of gender politics: “The question at the heart of the pronoun debate is fundamentally one of autonymy – the ability of a demographic, especially a marginalized one, to name itself and thus claim agency or control over how it is referred to, and by extension, treated” (53). Even though introduced in the discussion of pronouns, I suggest that “autonymy” can be extended to encapsulate this principle of nonbinary recognition as a privatization of gendered interpellation.

I see some antecedents for this principle of autonymy in earlier trans politics. The posttranssexual discourse of Stone and Stryker already privileges language as a political field from which to challenge gendered oppression, enacting a form of textualization of bodies. Berkins and Wayar’s travesti discourse reclaims a moment of individual autonomy and creativity, which does not demand an “alignment” between self-identification, naming, and gendered expression. What separates Young’s articulation of autonymy from the posttranssexual and travesti resignification is that the nonbinary subject is free of any contradiction, emerging as a consciousness untouched by the workings of gender. Second, Young’s autonymy politics are animated by a biopolitical desire to be governed by a state that appears in the narrative as a hopeful source of protection and validation against discrimination:

I do think it is important to discuss how many people identify as nonbinary or genderqueer (…). In this way, we can get a feel of the size of the nonbinary population and its geographic distribution (…). Accurate population estimates are essential for policymaking and the proper allocation of funds, resources, and public services. (Young, 2020, 33)

Young’s incorporation of a governmental logic in their political discourse contrasts with Stone and Stryker’s anti-foundationalist impetus and with the militant critique of the state as a source of violence in Berkins and Wayar’s discourse. Young’s project of autonymy takes hold of a utopian moment found in the posttranssexual and travesti projects but reintroduces an autonomous subject and limits its horizon to the Western liberal state.

### 3.5 Complicating nonbinary

However, Young’s autonymy project cannot be claimed to represent nonbinary discourse in general without ignoring other nonbinary authors. I consider Alabanza’s (2023) memoir “None of the Above” as a contrasting meaning of nonbinary discourse. The text chronicles and moves through a moment of crisis in Alabanza’s life and gender identity, concretized in the decision on whether to medically transition toward a more feminine appearance or not. By introducing Alabanza’s work, I want to show not only that very different political projects emerge from a nonbinary positionality, but also projects that align with Amin’s critique of identity and actualize a feminist critical theory tradition.

In Alabanza’s narrative, the meaning of nonbinary is not a concise definition but a dimension of Alabanza’s identity explored through a series of conflicts. I want to highlight three elements that contour a meaning of nonbinary distinct from Young’s. First, throughout the whole narrative, Alabanza’s use of nonbinary is inseparable from being visibly gender-non-conforming, that is, from gender self-expression that transgresses the norms of masculinity and femininity in recognizable ways. Second, Alabanza explicitly refuses a foundationalist understanding of trans and nonbinary identity. Alabanza writes, “I believe my transness is a reactionary fact, not an innate one. (…) I am trans because the systems the world operates through force me to be so, not because of genetics” (29). Finally, Alabanza claims nonbinary as an identity that interacts in complicated ways with class and race as further dimensions of their positionality (100). Recognition of nonbinary identities is, in Alabanza’s narrative, not an inherently positive act. For example, while nonbinary seems to hold a relevant critique of assimilationist transgender politics (50), it coexists with a “push for ‘nonbinary’ to be a legalized gender in the UK, which brings with it an attempt to homogenize and control what could have felt like a beautifully uncontrollable option” (103).

The overarching intervention I see in Alabanza’s text is the carving out of a discursive space to construct an account of medical transition that exposes the autonomous subject as an imposed fiction on the trans experience. Their struggle with the choice of medical transition stems in part from the impossibility of determining “who this is for” (183), emphasizing the difficulty of sustaining a notion of the self that is cordoned off from the social context in which the individual exists. Grappling with the choice of medical transition, Alabanza cannot find access to a sense of self and of pursuit that is not implicated in a vulnerability to the social circumstances in which we find ourselves:

As if ‘choosing what makes you happy’ is not related to the money you have in your bank account or could not affect the money potentially coming in. As if we exist as singular islands, where our choices for ourselves and our bodies are made in isolation from those around us, where we can pretend that each choice is only affected by or affects only us. (191)

Alabanza’s narrative thus struggles with gender autonomy as a political framework for gendered embodiment. On the one hand, nonbinary politics means “to fight for your own self, to advocate for your reality, and reclaim an autonomy over your body that was stripped from you at birth” (87). On the other hand, autonomy seems like an impossibility. Between one pole and the other, Alabanza crafts a complex meaning of autonomy. One that includes the choice to embody gender in a way that feels authentic and is worthy of respect, love, and care. Autonomy is not autarchy but is found, for instance, in the relational accomplishment of being “offered choice” (130). Alabanza’s narrative can thus be read in line with a critical notion of autonomy as “the ability to distance oneself from one’s social roles, traditions, history, and even deepest commitments and to take a universalistic attitude of hypothetical questioning toward them” (Benhabib, 1999, 353–354).

## 4 Neoliberal symptoms

In Amin’s take, nonbinary identities are a product of the neoliberal capture of gender politics. He argues that neoliberalism introduced a “universalization of identity as the basis of all politics that has made it appear necessary to announce one’s gender politics as an identity – nonbinary – rather than simply enacting them” (115f.). While it is unclear to me on what analysis Amin’s notion of neoliberalism as universalization of identity is based, I want to consider the claim that nonbinary discourse is symptomatic of neoliberalism: despite their marked divergences, is there a common thread between Alabanza’s and Young’s nonbinary narratives that can be read as a neoliberal symptom?

In thinking through this question, I am relying on Wendy Brown’s understanding of neoliberalism as “a peculiar form of reason that configures all aspects of existence in economic terms” and is “undoing basic elements of democracy” such as “vocabularies, principles of justice, political cultures, habits of citizenship, practices of rule, and above all, democratic imaginaries” (Brown, 2015, 17). In that regard, I read Young and Alabanza not as representatives of hegemonic Western liberal democracy, but as part of the traditions that condemn the insufficiency of this form of democracy, taking seriously Brown’s thesis that the impact of neoliberalism also extends to “radical democratic dreams” (*Ibid.*).

### 4.1 Resignifications of freedom and political belonging

I read Young’s notion of freedom and Alabanza’s figuration of political belonging as sites in which the neoliberal “hollowing out of contemporary liberal democracy and (…) imperiling of more radical democratic imaginaries” (*Ibid.*). Their figurations of freedom and political belonging rely on a split subject that is threatened by its fundamental social condition. This split subject can only arise as a credible emancipatory speaking position through the narrative construction of a condition of absolute political integrity, uncontaminated by oppressive meanings. This resignification of the emancipatory political subject as a pure subject has the effect of limiting the terrain of legitimate political conflict, which becomes circumscribed to shared identities and expressions of loyalty. By tracing these maneuvers in their narratives, I do not imply that Alabanza and Young cultivate antidemocratic desires, but that the meanings they articulate leave us less able to grasp and resist attacks on democratic values and institutions.

As I showed in the section on nonbinary autonymy, Young’s definition of nonbinary comes down to an individual act of gendered self-categorization, which establishes the primacy of individual meanings over the need for socially shared categories, which I define as a project of privatizing gendered interpellation. This understanding is, according to Young, “rooted conceptually in queer theory and the work of Judith Butler and Michel Foucault.” However, this influence “crystallized not in the classroom or in community meetings but on websites like LiveJournal and Tumblr” (Young, 2020, 8). This self-inscription in poststructuralist critique enables a reading of Young’s narrative as a resignification of this tradition in the figuration of nonbinary recognition as an escape from the violence of gendered interpellation. This imagination of freedom relies on an expansion of the meaning of violence that risks encompassing aspects inherent to the social condition of the subject. The desire to escape the violence inscribed in language that I see animating the strategy of autonymy, as understandable as it is, fails to acknowledge that it is “impossible to regulate fully the potential injurious effects of language without destroying something fundamental about language and, more specifically, about the subject’s constitution in language” (Butler, 1997, 27). The narrative of Young imagines an experience of freedom from violence that is threatened by the possibility of shared language, as well as by the blurring of boundaries between binary and nonbinary categories. The nonbinary subject of Young’s narrative identifies politics with the act of self-naming as the other of binary meanings. Butler’s critique of Wittig in Gender Trouble shows that such a strategy, in its contestation of gendered violence, constitutes politics in an identitarian logic that requires the disavowal of the other:

What a tragic mistake, then, to construct a gay/lesbian identity through the same exclusionary means, as if the excluded were not, precisely through its exclusion, always presupposed and, indeed, *required* for the construction of that identity (Butler, 1990, 174).

The combination of the desire to purify language of violence with the identitarian attachment inscribed in the project of nonbinary autonymy works toward a restriction of what is considered legitimate political conflict. The risk at work here is that, if one considers oneself to be defined as the other of oppression, dissent can never be legitimate, which makes me wonder what the place of democracy is in such politics.

This question also arises in an analysis of Alabanza’s construction of political community. As I argued, Alabanza’s nonbinary subjectivity emerges precisely in the claim that the notion of gender self-determination misrecognizes the social and political nature of gendered experience. This thinking enables the conception of a nonbinary subject beyond an identitarian logic. However, a close reading reveals a polarized separation at work in their narrative as well. At its core, the conflict that Alabanza grapples with is the betrayal of their younger self, who “would curse the me who just sat through excruciating pain each month to make sure I never have to shave again” (Alabanza, 2023, 8) and is projected onto the reader as holding a similarly intransigent gaze. In the narrative, Alabanza articulates all the ways in which being nonbinary or trans, medically transitioning or not, can be seen by the younger self or the reader as complicit with political projects of dominance in order to constitute themselves in radical opposition to those meanings. The conflict to be resolved is not whether to transition or not, but the construction of a non-complicit subjectivity in the decision to transition: “I will not know the answer, because to know an answer about something as illogical as gender is an impossible task, but I do promise to do it for us, for myself, and not for them” (209). The possibility of being seen as one of “them,” a category designating people complicit with oppressive institutions or norms, is constructed through Alabanza’s narrative as an existential threat. This reveals an identitarian understanding of political belonging and community that, in its radical implication, dissolves the terrain that would sustain a political dispute.

These narratives contrast with those of Stryker, Stone, Wayar, and Berkins, who work precisely by exposing the contradictions inherent in transsexual and travesti subjects. Part of their intervention is the self-critical examination of their implication in and attachment to violent structures and meanings. Rather than investing in the affirmation of a non-complicit standpoint, their critiques articulate the tension between the commitment to attend to the needs implicated in oppressive structures and the commitment to transcend these attachments through utopian critiques. I argue that this capacity to sustain internal contradictions and be torn apart in the process of articulating an emancipatory critique and a relevant political project is diminished in nonbinary narratives, constituting a symptom of neoliberal hollowing out of democratic cultures. However, if we assume the effects of neoliberal hegemony to be pervasive, on what basis can the current trans critic claim a voice untouched by neoliberal capture? This is also a personal question.

### 4.2 The construction of the trans critic

I have only been able to write this article after a long and exhausting process of undoing. For much of this time, I was attached to Amin’s analysis and sought to empirically substantiate a trans materialist critique of nonbinary identities as a problematic erasure of embodiment. Draft after draft was met with comments from colleagues and reviewers that pointed out inconsistencies and unclear points. Finally, I was able to discern and confront the ideological contradiction in which I had been caught. In the pretense of adjudicating the question of whether nonbinary identity claims can be seen as emancipatory or as neoliberal symptoms, the trans critic emerges as a subject outside of power^18^ and becomes a site of neoliberal capture of feminist critique and its democratic commitments. In showing the mechanism of this ideological operation in Amin’s article, I am engaging in my own repetition of his gesture under the sign of trans materialism.

First, Amin’s accusation of nonbinary discourse as erasing the social dimension of gender identities can be turned against his own analysis. The assessment of gender identities as abstract analytical propositions or overarching historical logics actively participates in the reification of gender identities by erasing the temporal, contextual, and conflicted dimensions of claiming nonbinary identities. To the extent that such a reification is a harm, it is one that Amin’s critique also commits by refusing to see gender identity claims as socially situated and open-ended political practices. Where Amin attacks nonbinary identities for having “no positive social content” (Amin, 2022, 117), it turns out that it is Amin’s approach itself that is not able to see gender identities in social terms. Not only is Amin’s approach unable to see the sociality in the phenomenon it seeks to analyze, but it also fails to recognize it in himself. His voice, unmarked by his own gender identity investments, resembles rather the “autological sovereign individual” of Western thought (116) who does not have to account for the social embeddedness of his subjectivity.

Second, Amin’s critique of a nonbinary subject becomes a vehicle for the construction of the trans critic as a split and authoritarian subject, an uncontaminated voice that issues a redemption narrative relying on the elimination of nonbinary identities. In this move, Amin constructs a false promise of political integrity that ignores “that there is no opposition of power which is not itself part of the very workings of power, that agency is implicated in what it opposes, that “emancipation” will never be the transcendence of power as such” (Butler, 1995b, 137). In this “urge to have philosophy supply the vision that will redeem life, that will make life worth living,” which Butler defines as “the very sign that the sphere of the political has *already* been abandoned” (Butler, 1995b, 131), it is possible to read the construction of the trans critic as another symptom of the hollowing out of democratic meanings under neoliberalism. In conclusion, a trans materialism that reduces the meaning of materialism to a positivist approach and ignores critical insights on the situatedness of theoretical practice reveals itself as an identitarian project in which the trans critic is constructed as a radical voice ultimately unable to establish connections to the terms of political struggle or acknowledge the contradictions or limits of their own narrative.

### 4.3 Trans materialism as theoretical practice

In conclusion, I argue that the real problem at stake is not the proliferation of nonbinary identities per se, but a tendency in nonbinary discourse, as well as in its trans critique, to undo democratic meanings, which is mediated by a loss in the transmission of feminist analysis as a self-critical practice. If we are concerned with the neoliberal capture of politics, we need to take seriously and examine the possibility that our critiques might be one more site of its unfolding. What follows from this is a task for the trans materialist turn: to actualize important resources in feminist theory for a self-critical, historical analysis of the constitution of the trans critic^19^. However, to the extent that we access these analyses through our present questions, desires, and premises, there is always the risk that we translate critical intentions and meanings into identitarian, undemocratic projects. This risk is especially acute when such translations take the form of redemption narratives that position our identity as the other of oppression.

What I want to emphasize is that the task of re-reading materialist theory from a trans political standpoint needs to go hand in hand with the cultivation of relations with dissenting others, including critical feminist traditions that are usually dismissed as not materialist. Without the many contestations from colleagues, but also without the empathy for nonbinary identified people in my environment, I would not have been pushed to delve into the contradictions of my attachment and ultimately transform my understanding of trans materialism in relation to feminist theory.

Considering the often polemical and antagonistic gestures present in some recent interventions in trans critique, I want to suggest that a contribution of the trans materialist turn might be to make visible the importance of relationality in our theoretical practice. This requires cultivating a tolerance for becoming vulnerable to the possibility of the breakdown of our theoretical and political identities within collective and coalitional attempts at transforming the conditions in which we are gendered. It also demands that we strengthen our consideration of multiple experiences of gendered oppression, defending the value of the fights people have fought in conditions they did not choose, as part of acknowledging that any fight for a better life can only be pursued in terms that feel relevant to people themselves. It is precisely in maintaining the commitment to both demands and holding space for the contradictions that necessarily emerge when we stop pursuing politics of purity that trans materialism can contribute to undermining the basis of reifications of gender identity and create new democratic habits.

I am aware that there are forms of dissent that represent existential threats in a life-threatening sense. The proposal to turn toward analysis that makes space for our contradictions and challenges our identities might seem an untimely idea under the current attacks on trans and nonbinary life. Probably, a more effective defense can be mounted on a reification of gender identity that can harness whatever credibility and power are left in the institutions of Western liberal democracy. However, if we want to imagine a world beyond the present, I believe it is essential to sustain a concept of resistance that includes the hope for impossible conversations.

## Data Availability

The original contributions presented in the study are included in the article/supplementary material, further inquiries can be directed to the corresponding author.

## Footnotes

^1^I would like to thank Hanna Meißner, Zoe* Steinsberger, Utan Schirmer, Aline Oloff and Sabine_ Hark for their support and critical feedback. I also want to express my gratitude to the anonymous reviewers for their constructive and thorough commentaries.

^2^For instance, Amin argues that “If, before heterosexuality, any normal man might have desired a fairy without any diminishment (…) of his manhood, now heterosexual men who are attracted to trans women may commit acts of extreme transmisogynist violence to protect their heterosexual masculine status. Extraordinary acts of transmisogynist violence may therefore be one consequence of the homo/hetero divide.” (111).

^3^“HIV-positive, thief, scandalous, infected, marginal” (all translations are from the author).

^4^“Is a complex interpellation to oneself, to society, to say: I am this; to what extent are you going to respect me?”

^5^“A way of seeing ourselves and being seen in a way that can enable or impede the recognition, enjoyment and access to rights.”

^6^“Traversed by the superficiality of the market.”

^7^“Our oppressor side.”

^8^“When you ask them in a workshop what they want to be, they answer: travesti. Or woman. They get trapped in that fictionality, and in this thing of being only transvestites. Only after a lot of work do other desires come out: teacher, dancer, doctor.”

^9^While we need to anchor our identity in some way in order to interpellate states as we seek positive inclusion policies, we must also be clear that in the everyday reality, identity is a concept that cannot be universalized or made uniform.

^10^“two-dimensional socialization.”

^11^“We are confronted in our culture by a number of common ideas that set related concepts in opposition to each other: mind/matter, intellect/body, nature/culture, subject/object, and theory/practice (…). It is obvious that ways of thinking that neglect interdependencies serve a social function whose problematic nature only becomes apparent through social critique.”

^12^“On the one hand, they must be understood as results of historical developments. In this sense, they are symptoms of divisions that destroy social interrelatedness. On the other hand, they are based on deception: they suggest unambiguity through cliché formation, while concealing the constructions that wrongly reconnect what has been polarized.”

^13^As book-length arguments, these texts allow for a more in-depth analysis of nonbinary discourse and politics than Amin’s anecdotal references to nonbinary celebrities, singular statistical survey, personal encounters and blog entries (Amin, 2022, 113–15).

^14^“An identity in flesh.”

^15^“We believe it is possible to construct our own gender, distinct from us.”

^16^“But even if I had not been able to undergo that transition, I would still be Lohana Berkins. Today I know that if I remove my breasts and cut my hair tomorrow, I will still be Lohana Berkins. We cannot think it’s only possible to be a travesti with that body.”

^17^As Adorno formulates in his introduction to sociology, positivist approaches follow a pragmatist logic that necessarily ends up fortifying the existing social systems (Adorno, 1993, 48f).

^18^The move to exempt oneself from the embeddedness within oppressive structures is neither new nor unique to trans politics. As one reviewer pointed out, Jasbir Puar offers a relatable analysis regarding the use of homonationalism as a moralizing accusation: “I myself do not think of homonationalism as an identity, a position, or an accusation — it is not another marker meant to cleave a “good” (progressive / transgressive / politically left) queer from a “bad” (sold-out / conservative / politically bankrupt) queer. (…) The accusation of homonationalism works to disavow our own inevitable and complex complicities with “queer” and with “nation.”” (Puar, 2017, 229–230).

^19^Following Fraser (2009), this would include a consideration of the shifts in the terrain in which trans and nonbinary identities and politics have been formed from the 1980s to the present, paying special attention to the synergies with the transformations of neoliberal capitalism. I want to point to some developments that surely would need to be taken into account such as the increased formalization and academization of feminist critique, the popularization of trans and queer culture, the uptake of trans and queer discourse by state institutions and corporate strategies, the establishment of the internet and social media as a social space for politicization and identity formation, formal depathologization of trans identities, but also, economic crisis, the successes of conservative, ethno-nationalist and anti-democratic political movements, their targeting of feminist, queer and trans culture and politics as major threat, and their uplifting of a specific feminist anti-trans discourse with unprecedented social reach.
